# Photosynthesis-dependent formation of convoluted plasma membrane domains in *Chara* internodal cells is independent of chloroplast position

**DOI:** 10.1007/s00709-014-0742-9

**Published:** 2014-12-19

**Authors:** Ilse Foissner, Aniela Sommer, Margit Hoeftberger

**Affiliations:** Plant Physiology/Cell Biology, University of Salzburg, Hellbrunnerstrasse 34, 5020 Salzburg, Austria

**Keywords:** Charasome, *Chara australis*, pH banding, Plant, Nodal cell, Wound

## Abstract

The characean green alga *Chara australis* forms complex plasma membrane convolutions called charasomes when exposed to light. Charasomes are involved in local acidification of the surrounding medium which facilitates carbon uptake required for photosynthesis. They have hitherto been only described in the internodal cells and in close contact with the stationary chloroplasts. Here, we show that charasomes are not only present in the internodal cells of the main axis, side branches, and branchlets but that the plasma membranes of chloroplast-containing nodal cells, protonemata, and rhizoids are also able to invaginate into complex domains. Removal of chloroplasts by local irradiation with intense light revealed that charasomes can develop at chloroplast-free “windows” and that the resulting pH banding pattern is independent of chloroplast or window position. Charasomes were not detected along cell walls containing functional plasmodesmata. However, charasomes formed next to a smooth wound wall which was deposited onto the plasmodesmata-containing wall when the neighboring cell was damaged. In contrast, charasomes were rarely found at uneven, bulged wound walls which protrude into the streaming endoplasm and which were induced by ligation or puncturing. The results of this study show that charasome formation, although dependent on photosynthesis, does not require intimate contact with chloroplasts. Our data suggest further that the presence of plasmodesmata inhibits charasome formation and/or that exposure to the outer medium is a prerequisite for charasome formation. Finally, we hypothesize that the absence of charasomes at bulged wound walls is due to the disturbance of uniform laminar mass streaming.

**Electronic supplementary material:**

The online version of this article (doi:10.1007/s00709-014-0742-9) contains supplementary material, which is available to authorized users.

## Introduction

The plasma membrane of plant and other cells is laterally compartmented into a wide range of different types of domains. Among these domains, lipid rafts are considered to be small (10–200 nm in diameter) and highly dynamic (Lingwood and Simons 2009; Mongrand et al. 2011), whereas micro- and macrodomains are larger (up to several micrometers) and more stable (Boutte and Moreau 2014; Jarsch et al. 2014; Malinsky et al. 2013). Membrane domains have been shown to be involved in signal transduction, cell to cell interactions, transport, stress, and polarized growth. They are enriched or depleted in membrane components but structurally similar to the “normal” plasma membrane. Visually more conspicuous are highly convoluted plasma membrane areas which serve to provide space for the accommodation of various transporters, e. g., the microvilli of animal intestinal cells (Lange 2011) or the convoluted plasma membrane domains of plant transfer cells (Offler et al. 2003). Convoluted membrane domains are also characteristic for submerged leaves of aquatic angiosperms where the plasma membrane along the outer epidermal cells is invaginated along distinct wall ingrowths in order to provide space for the accommodation of H^+^-ATPases (Elzenga and Prins 1989a, b; Ligrone et al. 2011; Prins and Elzenga 1989; Prins et al. 1982). The H^+^-ATPases acidify the environment which helps to convert membrane-impermeable hydrogen carbonate (bicarbonate) into CO_2_ which is freely diffusible and easily taken up for photosynthesis. Plasma membrane domains with a similar structure and a similar function are present in the highly evolved characean alga, *Chara australis*, as described below.

**Fig. 1:**
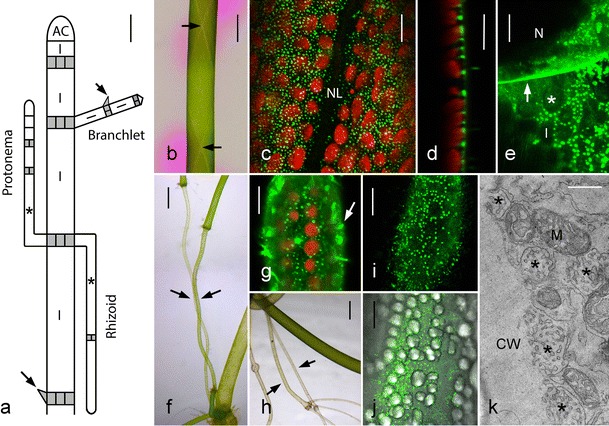
Thallus of *Chara australis* and charasomes in internodal cells. **a** Simplified schematic drawing of a *Chara* thallus. The apical cell (*AC*) gives rise to a regular series of cylindrical internodal cells (*I*) and groups of nodal cells (*gray*). Nodal cells may divide further and produce side branches (shoots of unlimited growth like that of the main axis; not shown) and branchlets (shoots of limited growth). Nodes incubated in darkness produce upward growing protonemata and downward growing rhizoids. Protonema and rhizoid internodal cells investigated in this study are marked by *asterisks*. Non-dividing cone-shaped cells are called stipulodes (*long arrow*) in nodes of the main axis or bract cells (*short arrow*) in nodes of branchlets. **b** Light micrograph of an internodal cell incubated in phenol red. *Pink color* indicates alkaline pH. The arrows point to the intersections of chloroplast-free neutral lines located at the upper and lower surface of the cells. **c** Cortex of internodal cells containing helical files of stationary chloroplasts (*red autofluorescent*) and FM1-43-stained charasomes (*green fluorescent*). Note absence of charasomes at the neutral line (*NL*). **d** Side view of charasomes (*FM1-43-fluorescent*) between chloroplasts (*red autofluorescent*). **e** The *green fluorescent* FM1-43-stained plasma membrane at the cross wall (*arrow*; optical cross-section) between a nodal (*N*) and an internodal cell (*I*) is smooth (compare with (**d**)). In the internodal cell charasomes are seen at the longitudinal wall and between the chloroplasts (empty spaces; *asterisk*). Plasmodesmata are out of focus. **f** Two intertwining basal protonema internodal cells (*arrows*). **g** *Red fluorescent* chloroplasts and *green fluorescent*, FM1-43-stained punctate charasomes in a basal protonema internodal cell. The arrow points to FM1-43-stained epiphytes. **h** Proximal internodal cells of rhizoids (*arrows*) after 2 weeks of exposure to light. **i**–**k** Charasomes in a proximal rhizoid internodal cell. CLSM image of FM1-43-stained fluorescent charasomes (**i**) between starch-containing chloroplasts visualized by DIC in the merged image (**j**). The electron micrograph (**k**) is a slightly tangential section showing charasomes (*asterisks*) along the cell wall (*CW*) and mitochondria (*M*). *Bars* = 1 mm (**a**, **b**, **f**, **h**), 20 μm (**e**, **i**, **j**), 10 μm (**c**, **d**, **g**), and 500 nm (**k**)

The characean thallus consists of a regular series of long, cylindrical internodal cells and groups of small, isodiametric or flat nodal cells from which side branches (shoots with unlimited growth) and whorls of lateral cells (branchlets, shoots with limited growth) emerge (Fig. 1a) (Wood and Imahori 1965). Growth occurs by the ordered division of a dome-shaped apical cell, which sequentially produces the internodal and nodal cells. Sexual reproduction involves the germination of an oospore, which produces a positively gravitropic primary rhizoid and a negatively gravitropic, upward growing primary protonema from which a new thallus emerges. Secondary protonemata and secondary rhizoids arise from nodes isolated from the differentiated thallus and buried in the sediment (Braun and Limbach 2006; Hodick 1993).

The internodal cells of the characean algae may become up to 20 cm long. They have been a widely used model system in plant biology and are useful for studying various aspects of cellular dynamics and the cytoskeleton (Foissner and Wasteneys 2012; Shimmen and Yokota 2004). They are also well suited for the study of transmembrane transport and plasma membrane domains (Beilby and Bisson 2012; Beilby and Casanova 2014; Foissner and Wasteneys 2014; Volkov 2006). The cytoplasm of the internodal cells consists of a stationary cortex, which includes files of helically arranged chloroplasts (Fig. 1b, c), a motile endoplasm, and a huge central vacuole. Cytoplasmic rotational streaming is generated by interaction of myosin-coated organelles with subcortical actin bundles attached to the inner surface of chloroplasts. Up- and down-streaming regions are separated from each other by the chloroplast-free neutral line (Fig. 1b, c). The plasma membrane along the longitudinal cell walls is smooth in the internodal cells of the genera *Nitella* and *Nitellopsis* as investigated so far. In the internodal cells of the genus *Chara*, however, smooth plasma membrane domains alternate with highly complex convoluted membrane areas in light-exposed thalli (Chau et al. 1994; Franceschi and Lucas 1980; Schmoelzer et al. 2011 and references therein). The function of these “charasomes” is similar to that of the membrane invaginations found in leaves of submerged water plants (see above). Several studies indicate that they are involved in the photosynthesis-dependent acidification of the environment required for efficient uptake of CO_2_ (e.g., Chau et al. 1994; Price and Badger 1985; Price et al. 1985; Price and Whitecross 1983). A recent study confirmed the abundance of H^+^-ATPases in charasomes and their involvement in the generation of the pH banding pattern at the cell surface under steady state conditions (Schmoelzer et al. 2011). However, it has also been shown that a pH banding pattern can be generated in the absence of charasomes, and that photosynthetic utilization of exogenous hydrogen carbonate can occur in the absence of charasomes (e.g. Lucas et al. 1989). Unlike the convoluted plasma membrane areas in cells of higher plants (McCurdy et al. 2008), charasomes are not supported by cell wall ingrowths but by a proteoglucan coat (Franceschi and Lucas 1981). This allows easy degradation and, eventually, reformation at other cell regions in response to changing light conditions (Schmoelzer et al. 2011). So far, charasomes have been described only in the internodal cells of the main axis, the branches and the branchlets, and in close proximity to the chloroplasts. They have been reported to be absent from the chloroplast-free neutral lines and from the chloroplast-free cross walls (Franceschi and Lucas 1980; Schmoelzer et al. 2011). On the basis of these data, it was concluded that the induction of a (charasome-free) alkaline zone at regions ligated with the aid of cotton or silk thread was due to the removal of the chloroplasts (Shimmen and Yamamoto 2002).

During the course of this study, we wanted to verify or discard the hypothesis that the formation of charasomes requires intimate contact of chloroplasts with the plasma membrane, and that the local experimental removal of chloroplasts from the cell cortex results in degradation of charasomes and, consequently, the formation of an alkaline zone. Our data show that charasomes can develop at chloroplast-free windows produced by local irradiation with intense light and that the pH banding pattern is independent of these chloroplast-free areas. Our study revealed also that the presence of functional plasmodesmata excludes the presence of charasomes, and that charasomes are rarely formed at wound walls which protrude into the streaming endoplasm. We show further that charasomes are not only formed in the internodal cells of the main axis and the branches, but that the plasma membrane of the nodal cells, protonemata, and rhizoids is also able to invaginate into these complex membrane domains.

## Material and methods

### Algal material and culture conditions

The thalli of *Chara australis* were grown in a substrate of soil, peat, and sand in 10–50 l aquaria filled with distilled water. The temperature was about 20 °C and fluorescent lamps provided a 16/8 h light/dark cycle. The light intensity was low (about 5 μEinstein m^−2^ s^−1^) in order to prevent the calcification and excessive growth of epiphytes. After several weeks of growth, fragments of thalli were isolated from the main axis with a small pair of scissors and left in artificial fresh water (AFW) (10^−3^ M NaCl, 10^−4^ M KCl, 10^−4^ M CaCl_2_) until use.

For growth of protonemata, shoots were cut into short segments containing at least two nodes and one internodal cell as described (Braun 2002). The segments were embedded in a 1-cm-high layer of sand which covered the bottom of a glass container measuring about 6 cm in diameter and about 6 cm in height. The containers were filled with slightly modified Forsberg medium (Forsberg 1965), supplemented with 1 % soil (*w*/*v*), and kept in complete darkness. After 3 to 4 weeks, the single-celled, pale protonema initials had a length of up to 2 cm. These protonema initials started cell divisions when exposed to light, and the basal cell became green after a few days (Fig. 1a).

### In vivo staining, formation of chloroplast-free windows and confocal laser scanning microscopy

The pH banding pattern of the internodal cells was documented using phenol red (phenolsulfonphthalein, Sigma) at a concentration of 10 mM dissolved in AFW. Phenol red changes color from yellow to pink depending on the pH of the medium (visual transition interval is between pH 6.8 and pH 8.2; Spear et al. 1969). Photographs were taken after 15 min exposure to intense light of fluorescent lamps (80 μEinstein m^−2^ s^−1^).

For in vivo staining of charasomes, internodal cells were pulse labeled for 5 min with 10 μM FM1-43FX (N-(3-triethylammoniumpropyl)-4-(4-(dibutylamino)styryl)pyridinium dibromide, Invitrogen, Carlsbad, CA, USA), a plasma membrane dye and endocytic marker, diluted from a 500 μM stock solution in AFW. Mitochondria were fluorescently labeled by 1 µM MitoTracker® Orange CMTMRos (Invitrogen) diluted from a 1 mM stock solution in DMSO.

The chloroplast-free windows in the internodal cells of the main axis and the branchlets were created by 3–7 min irradiation of cells with the blue light (450–490 nm) of a halide lamp (maximum intensity) attached to a fluorescence microscope and using a 40× objective (Kamitsubo 1972). This treatment was sufficient to cause rounding up of chloroplasts and to temporarily impede streaming in the irradiated zone. Longer irradiation times which lead to the deposition of a wound wall (Foissner and Wasteneys 2012) were not applied. For recovery and for charasome formation, cells were placed into Petri dishes containing AFW or a mixture of AFW and modified Forsberg medium and exposed to light provided by fluorescent lamps with an intensity of about 5 μEinstein m^−2^ s^−1^. The bleached chloroplasts were detached from the cortex within 2 hours and were carried away with the streaming endoplasm. The pH banding pattern and charasome distribution were documented at various time intervals as described above.

The confocal laser scanning microscope (CLSM) used in this study was a Leica (Mannheim, Germany) TCS SP5 coupled to a DMI 6000B inverted microscope. Live internodal cells and nodal complexes were mounted in AFW ± dye. All images presented in this study are single optical sections unless otherwise stated. Images produced by the LSM software were further processed with Adobe Photoshop (Adobe Systems Inc.).

### Electron microscopy

Chemical fixation was as described in Foissner (1991). Briefly, cells were fixed for 30 min at room temperature in 1 % glutaraldehyde dissolved in phosphate buffer, pH 6.8. Following several washes in buffer, cells were postfixed overnight at 4° in 2 % OsO_4_ dissolved in buffer. After dehydration in an ethanol series at 4 °C, cells were embedded in Agar low viscosity resin (Agar Scientific, Essex, Great Britain). Micrographs at elastic bright-field mode were taken with a Zeiss LEO 912 transmission electron microscope (EM) equipped with in-column energy filter.

## Results

### Charasomes in the internodal cells of the main axis, branches, branchlets, protonemata and rhizoids

Fluorescent plasma membrane dyes like FM1-43 accumulate in the membranous tubules of charasomes and can therefore be used for visualization of charasomes in living cells (Schmoelzer et al. 2011). The FM-dyes also stain membrane- and callose-containing amorphous wound walls deposited after injury or in senescent cells (Foissner and Wasteneys 2012; see below). In order to exclude the possibility that small wound walls were mistaken for charasomes, we used electron microscopy and/or co-labeling with sirofluor, a callose-specific fluorescent stain which labels the wound walls only (see Klima and Foissner 2008). Experiments with pH buffers and with inhibitors of H^+^-ATPase activity like vanadate indicate that FM1-43 staining of charasomes is neither dependent on the pH nor on their functional status (Suppl. Fig. 1; see also Schmoelzer et al. 2011).

The possibility to stain charasomes in living cells, with the aid of fluorescent plasma membrane and endocytic markers (Klima and Foissner 2008; Schmoelzer et al. 2011), confirmed the earlier electron microscopical studies about the presence of charasomes in the internodal cells of the main axis, the branches, and the branchlets (Fig. 1a; Bisson et al. 1991; Chau et al. 1994; Franceschi and Lucas 1980 and references therein). Under steady state conditions, charasome size and abundance correlated with the pH banding pattern as revealed by the pH indicating dye phenol red (Fig. 1b). Charasomes were large (up to few micrometer in diameter) and numerous at the acid regions (Fig. 1c, d), and they were smaller, less abundant, or even absent at the alkaline zones (not shown) as described in detail in Schmoelzer et al. (2011). Mean charasome area fractions (percentage of cortical area occupied by charasomes) in cells investigated during the course of this study were 18.7 (±1.9 SD, *n* = 6) at the acid regions and 2.2 (±0.2 SD, *n* = 5) at the alkaline zones, and differences were highly significant (*P* ≤0.001, *t* test). Transitions from acid to alkaline pH were smooth or abrupt (Bulychev et al. 2003) and correlated with smooth or abrupt changes in charasome size and abundance (Schmoelzer et al. 2011). Consistent with earlier findings (Franceschi and Lucas 1980), charasomes were found to be absent from the chloroplast-free neutral line (Fig. 1b, c) and from the chloroplast-free cross walls (Fig. 1e). In this study, we additionally investigated the more delicate basal (proximal) internodal cells of the secondary protonemata (Fig. 1a, f) and the upper (proximal) internodal cells of the rhizoids (Fig. 1a, h). Their cytoplasmic architecture is similar to that of the internodes of the main axis and the branchlets consisting of a stationary cortex, a streaming endoplasm which contains up to several thousand nuclei and a large central vacuole. In the upward-growing green protonema, the internodal cell charasomes were detected by CLSM of FM1-43 stained cells (Fig. 1g) and by the electron microscopy (data not shown). Freshly collected rhizoid internodal cells never contained charasomes. However, when these pale rhizoids were pulled out of the sediment and exposed to light for about 2 weeks, numerous charasomes could be identified by CLSM (Fig. 1i, j) and EM (Fig. 1k). In protonemata and rhizoids, charasomes often formed in considerable distance to the small and widely spaced chloroplasts (Fig. 1g, j). The abundance of charasomes in the internodal cells of protonemata and rhizoids was not uniform suggesting uneven acidification of the cell surface. A pH banding pattern, however, could not be detected using phenol red. Most probably, the differences in acid and alkaline pH were too small to be detected by pH-indicating dyes. Neither chloroplasts nor charasomes were detected in the tip-growing cells of protonemata or in tip-growing rhizoids.

### Charasomes in nodal cells

**Fig. 2:**
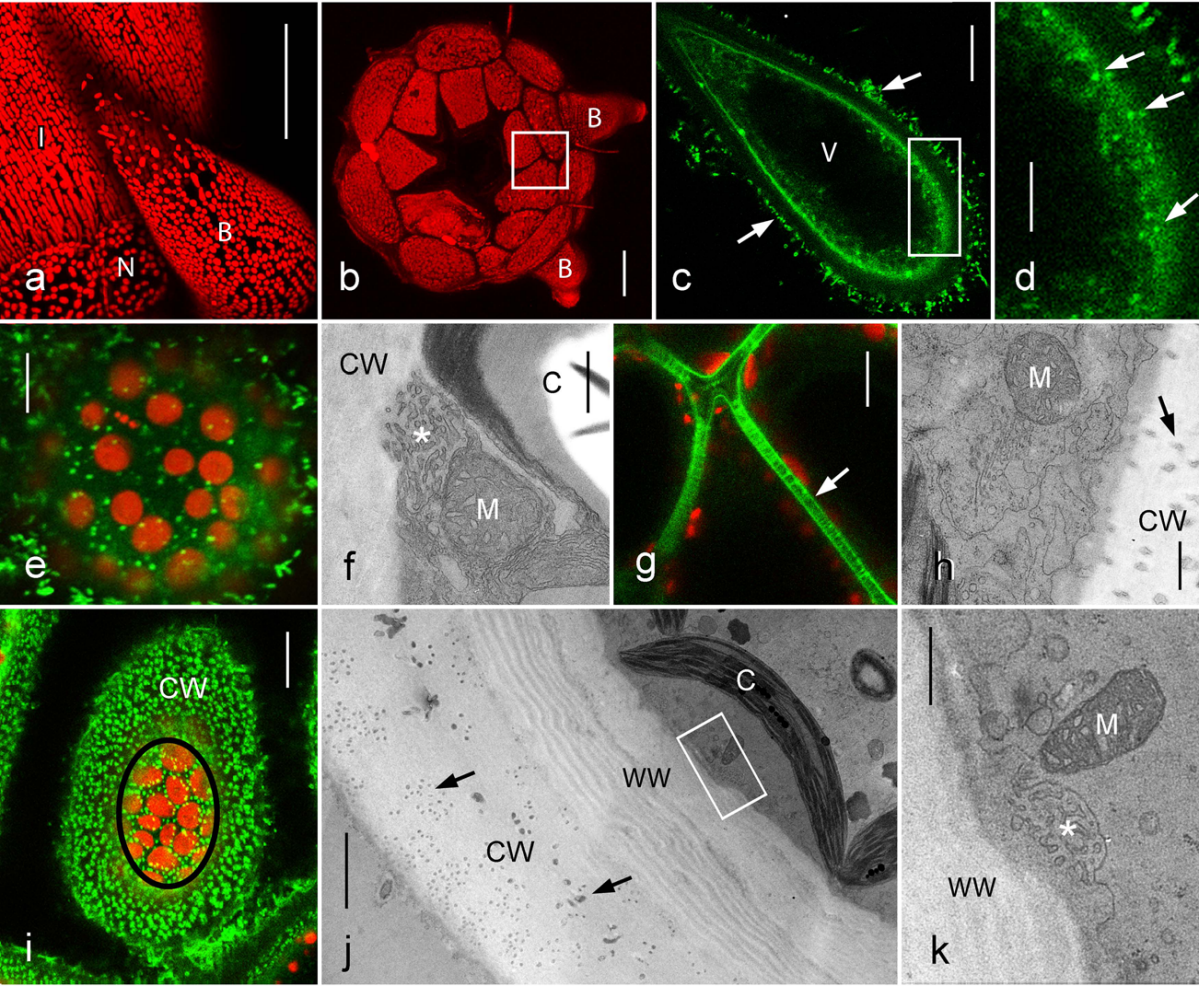
Chloroplasts and charasomes in nodal cells. **a** Side view of nodal complex and adjacent internodal cell (*I*) visualized by fluorescent chloroplasts (projection of 109 optical sections with a thickness of 1.4 μm). Peripheral roundish nodal cells (*N*) alternate with bract cells (*B*). **b** Autofluorescent chloroplasts in a nodal complex viewed from above and after removal of internodes (projection of 134 optical sections with a thickness of 1.4 μm). The central, flat nodal cells (several cells died during preparation) are surrounded by peripheral, roundish nodal cells which intercalate with cone-shaped bract cells (*B*). The inset corresponds to Fig. 2g. **c**, **d** Optical sections through the periphery of an FM1-43-stained bract cell. *Arrows* indicate fluorescent epiphytes at the cell wall, *V* is the central vacuole. *Arrows* in the higher magnification (**d**) show immobile charasomes near the cell wall. Mobile endosomes are seen near the vacuole. **e**, **f** Charasomes and chloroplasts at the outer cell wall of peripheral nodal cells. The fluorescence image (**e**) shows autofluorescent chloroplasts (*red*) and FM1-43-stained charasomes (*green*). The electron micrograph (**f**) shows a charasome (*asterisk*) beneath the cell wall (*CW*) and close to a chloroplast (*C*) and to a mitochondrion (*M*). **g**, **h** Plasma membrane and plasmodesmata along the inner cell walls of nodal cells. The *arrow* in the fluorescence image (**g**) points to a cell wall pervaded by plasmodesmata which were stained with green fluorescent FM1-43. Note absence of charasomes; several chloroplasts (*red autofluorescent*) are present in the cortical cytoplasm. The *arrow* in the electron micrograph (**h**) points to a plasmodesmal channel in the cell wall (*CW*). The plasma membrane is wrinkled due to chemical fixation but charasomes are absent. **i**–**k** Charasomes at the inner cell wall of nodal cells formed after removal of internodal cells. In the fluorescence image (**i**) the *green* FM1-43 fluorescent structures in the cell wall (*CW*) correspond to channels containing the damaged plasmodesmata. In the cytoplasm (*encircled*) FM1-43 stained charasomes (*green*) are seen between autofluorescent chloroplasts (*red*). The electron micrographs (**j**) and (**k**) show that a thick wound wall (*WW*) had been deposited over the cell wall (*CW*) which is pervaded by plasmodesmata-containing channels (*arrows* in (**j)**, *C* represents chloroplast, the inset corresponds to Fig. 2k). The higher magnification (**k**) shows a charasome (*asterisk*) beneath the wound wall (*WW*) and close to a mitochondrion (*M*). *Bars* = 100 μm (**a**, **b**), 20 μm (**c**, **g**, **i**), 10 μm (**d**, **e**), 2 μm (**j**), 500 nm (**f**, **h**, **k**)


Nodal cells differ from the internodal cells in their shape, size, and in the presence of only one nucleus (Beilby and Casanova 2014; Wood and Imahori 1965). Apart from this very general definition, nodal cells vary considerably in localization, shape, and cytoplasmic organization (Figs. 1a and 2a, b). The nodes of *Chara australis* consist of a group of flat central cells squeezed between the upper and lower internodal cell. The flat central nodal cells are surrounded by peripheral, roundish cells intercalating with cone-shaped cells called stipulodes or bract cells dependent on whether they are located at the nodes of the main axis or the nodes of the branchlets (Figs. 1a and 2a, b). No charasomes were found in nodes of freshly collected, growing thalli, which were cultivated at rather low light intensities (about 5 μEinstein m^−2^ s^−1^ at the surface of culture vessels). In fully grown thalli and in thallus fragments exposed to stronger light, FM1-43 fluorescent immobile punctae were detected in the cortical cytoplasm of stipulodes and bract cells (Figs. 2c, d and 5) and the electron microscopy confirmed their identity as charasomes (not shown). The cytoarchitecture of these cells mostly resembled that of the internodes and consisted of a stationary layer of well-developed chloroplasts and a streaming endoplasm. Charasomes were also present in the peripheral roundish nodal cells (Figs. 2e, f and 5) irrespective whether their cytoplasm was stagnant or separated into a stationary cortical layer and a streaming endoplasm. Occasionally, we observed nodal cells in which chloroplasts participated in rotational mass streaming and even in these cell charasomes, although very delicate ones, could be detected (Suppl. Video 1). A prerequisite for charasome formation appeared to be the presence of chloroplasts because charasomes were never detected in those peripheral nodal cells, which were likely to give rise to a side branch and contained only proplastids embedded in a dense, non-streaming cytoplasm.

Nodal cell of *Chara australis* with motile chloroplasts (autofluorescent red) and minute FM1-43 fluorescent charasomes (green; arrows). Time interval is2580 ms; 5 frames per second. (AVI 842 kb)


In freshly isolated nodes, the charasomes in peripheral nodal, stipulode or bract cells were exclusively located along their outer cell walls, i. e., at those facing the environment (Figs. 2c–e and 5). No charasomes could be detected at the plasmodesmata-containing cell walls, i. e., at regions with close contact to another cell (an internode or a cell of the nodal complex). The central flat nodal cells (Fig. 2b and schematic Fig. 5), which were completely surrounded by the peripheral nodal cells and by the upper and lower internodes, were therefore completely free of charasomes (Figs. 2g, h and 5). In the nodal cells, chloroplasts were seen not only at the plasmodesmata-free but also at the plasmodesmata-pervaded cell walls (Figs. 2a, b, g and 5), which is in contrast to the internodal cells where neither charasomes nor chloroplasts could be detected at the plasmodesmata-containing cross walls (Figs. 1e and 5).

However, charasomes formed beneath the plasmodesmata-containing cell walls when nodal complexes were isolated from the thallus as shown in Fig. 2b and were exposed to light (16/8 h light/dark cycle, 5 μEinstein m^−2^ s^−1^) for at least 2 weeks. Following staining with FM1-43, numerous tubular structures became visible in those cell walls, which previously connected the nodal cells with their adjacent internodes (Fig. 2i). These tubules corresponded to the channels containing the damaged plasmodesmata. Their brighter FM1-43 fluorescence, as compared with that of the living plasmodesmata, suggests that FM1-43 stained not only the residual plasma membrane but also other components of plugged plasmodesmata (compare Figs. 2g with 2i). The cytoplasm beneath these cell walls contained FM1-43-stained, immobile punctate structures in the interstices between the cortical chloroplasts (Fig. 2i). Electron microscopy revealed that these charasomes were not located immediately beneath the original, perforated cell wall but beneath a channel-free, smooth wound wall deposited in response to the severing of plasmodesmata (Figs. 2j, k and 5).

### Charasomes and pH banding in chloroplast-free windows

**Fig. 3:**
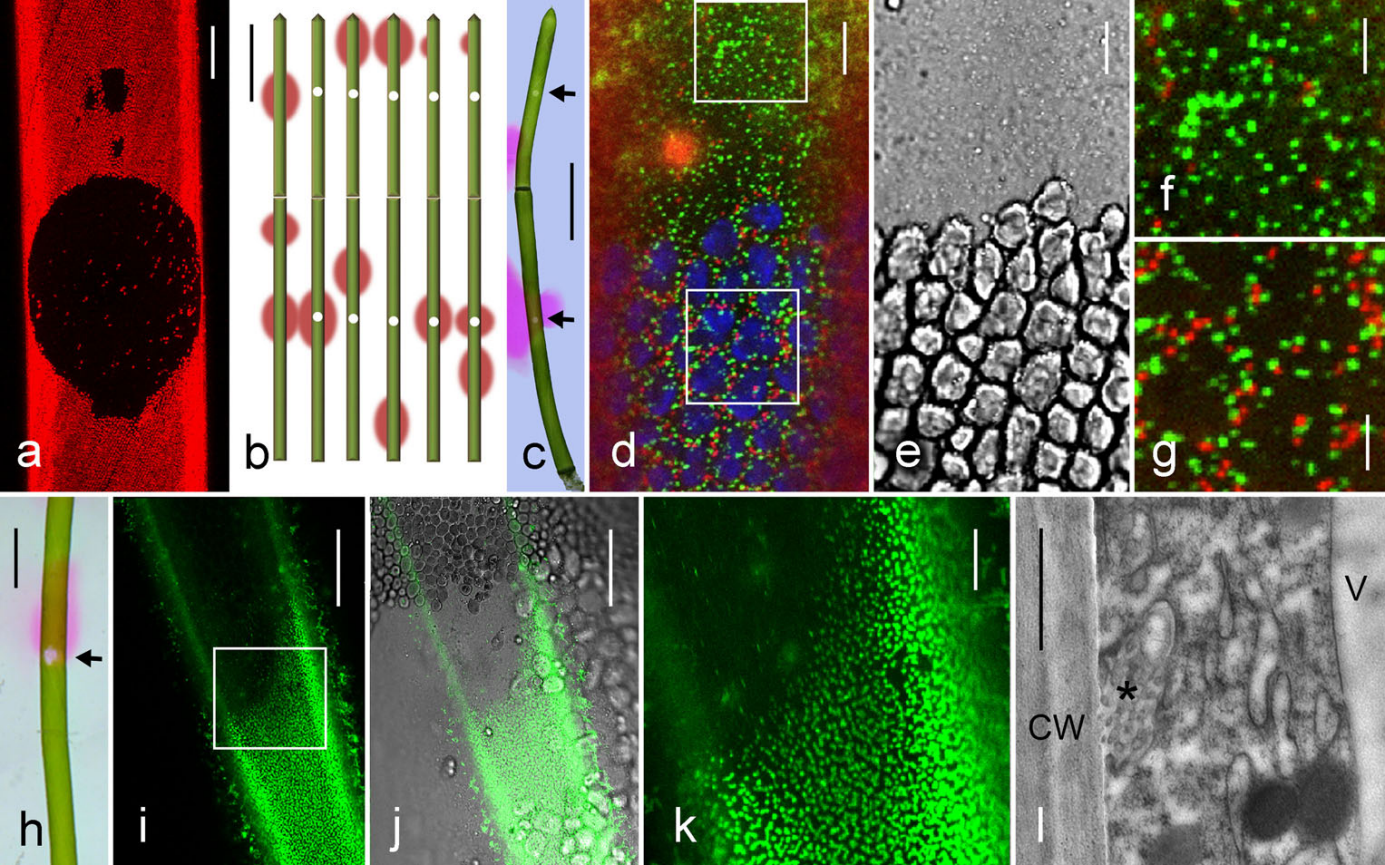
Charasomes in chloroplast-free windows. **a** Chloroplasts (*red fluorescent*) in an internodal cell of the main axis are absent from windows created by irradiation with blue light (projection of 146 optical sections with a thickness of 1.4 μm). **b**, **c** Schematic drawing and light micrograph showing the pH banding pattern visualized by phenol red in two neighboring internodal cells of a branchlet before and in 1-week intervals after the creation of windows (*white circles*) at the alkaline bands (*pink*). Note that the upper window became and remained acidic whereas the pH at the window in the lower cell was variable. In the light micrograph (**c**) the positions of the windows are indicated by *arrows*. **d**–**g** Charasomes stained by *green fluorescent* FM1-43 and mitochondria labeled with *red fluorescent* MT orange in an acidic window and in the adjacent control region. Charasomes are more evenly distributed in the window than between the autofluorescent chloroplasts (false *colored blue* in (**d)**). Mitochondria are less abundant in the window. **e** The corresponding DIC image. **f**, **g** Higher magnifications corresponding to the insets shown in (**d**). **h**–**k** Charasome distribution in a window in which the pH abruptly changes from alkaline to acidic (*arrow* in (**h**)). The acidic part of the window contains abundant and large charasomes stained by *green fluorescent* FM1-43; in the alkaline part of the window charasomes are absent. The fluorescent image merged with the bright field image (**j**) and a higher magnification (**k**) of the inset shown in (**i**). **l** Electron micrograph of a charasome (*asterisk*) near the cell wall (*CW*) of a chloroplast-free window; *V* is the central vacuole. *Bars* = 1 cm (**b**, **c**, **h**) 200 μm (**a**), 50 μm (**i**, **j**), 10 μm (**d**, **e**, **k**), 5 μm (**f**, **g**), 500 nm (**l**)

Our observations showed that, firstly, charasomes often developed in considerable distance to the stationary chloroplasts in protonema and rhizoid cells and that, secondly, charasomes were present in nodal cells with mobile chloroplasts. These findings prompted us to investigate whether and how the experimental removal of chloroplasts in the internodal cells affected charasome formation and pH banding. We therefore produced chloroplast-free “windows” by local irradiation of cells with the intense light of a halide or mercury lamp attached to a microscope. For our experiments, we isolated non-calcified and non-growing internodal cells and incubated them in artificial fresh water for 1 day. The pH banding pattern of these cells was variable over a period of several days consistent with earlier observations in the internodal cells without CaCO_3_ deposits (Suppl. Fig. 2; Lucas and Smith 1973). Nonetheless, the pH banding pattern largely correlated with the size and density of charasomes as described above; i. e., they were large and abundant at the acid regions and small and scarce at the alkaline areas. We also investigated the internodal cells which had been incubated in darkness for 2 weeks in order to degrade the charasomes. These cells likewise produced acid and alkaline bands, although they contained no or only very few, small charasomes which were evenly distributed along the longitudinal cell walls (Bisson et al. 1991; Schmoelzer et al. 2011). The results obtained with the internodal cells of the branchlets were similar to those obtained with the internodal cells of the main axis and independent of the size of windows which had a diameter between 60 and 400 μm. In the following, we describe windows in the internodal cells of branchlets and with a size that corresponded to about one-third of the cell circumference (about 200 μm, Fig. 3a). Information about pH banding at chloroplast-free windows in the internodal cells of the main axis are found in Suppl. Fig. 2.

We produced windows at the alkaline or at the acid regions previously identified by incubation in phenol red. Local irradiation with intense blue light caused local, transient alkalinization of the medium as reported from microperforated internodes (Bulychev and Komarova 2014b) even if windows were created at the acidic zones (not shown). After window formation, the pH banding patterns were documented in 1 week intervals over a period of up to 5 weeks. As illustrated in Fig. 3b, c, the pattern of acid and alkaline regions changed within days similar to that of control cells without windows. Some alkaline regions, especially those at the tip of the distal internodal cells of the branchlets were rather stable, others disappeared or slightly changed their position and/or size, and still others seemed to form de novo at the previously acid bands. No correlation was found between the positions of windows and the positions of alkaline or acid bands, irrespective whether windows were created at the acid or at the alkaline zones, and irrespective whether charasomes were present or not. Over a period of 5 weeks, 22 out of 34 windows became permanently acidic, 10 windows became transiently alkaline, and 2 windows became permanently alkaline.

Just as the pH banding pattern, the distribution of charasomes, documented between 1 and 2 weeks after local removal of chloroplasts, was independent of the position of windows. No or very few charasomes were found at alkaline windows even if the windows were created at acid (charasome-rich) zones (not shown). Charasomes were larger and more abundant in windows with an acidic pH. When the window was located within a larger acid region, charasome size and abundance within the window were similar as in the chloroplast-containing neighbor area, but charasome distribution was different (Fig. 3d–g). Outside the window, charasomes were preferentially located in the clefts between chloroplasts (Fig. 3g), but they were evenly distributed in the chloroplast-free areas (Fig. 3f). When the border between an acid and alkaline zone was located across a window, transitions in charasome size and abundance were either continuous or abrupt as reported for control cells (Schmoelzer et al. 2011). Abrupt transitions yielded the most spectacular images. In these windows, the extremely dense pattern of large, punctate, or branched charasomes in the acid region sharply contrasted with the complete absence of charasomes at the adjacent alkaline zone (Fig. 3h–k).

Under steady state conditions, the pH banding pattern correlated not only with spatiotemporal differences in charasome size and abundance but also with spatiotemporal differences in size and abundance of cortical mitochondria, which were larger and more numerous at the acid regions as compared with the alkaline bands (Foissner 2004; Schmoelzer et al. 2011). Within windows, however, cortical mitochondria were rarely observed, even if they had an acidic pH and a dense population of charasomes (Fig. 3f, g). Inside the windows, mitochondria performed wiggling motions and often became detached and were carried away with the streaming endoplasm. On the other hand, endoplasmic mitochondria were observed to suddenly establish contact with the cortex and to remain attached for several seconds or minutes. The mitochondria in windows were often streaming aligned, i. e., parallel to the direction of cytoplasmic streaming (not shown). Streaming-aligned orientation of charasomes was never observed.

### Charasomes and pH banding at wound walls

It has been reported that alkaline bands form at wounds induced, e.g., by ligation of the internodal cells, and it had been suggested that this local alkalinization was due to the removal of chloroplasts (Shimmen and Yamamoto 2002). In this study, we were able to demonstrate that the mere absence of chloroplasts did not automatically lead to a permanent alkalinization of the cell surface even when the chloroplast-free area occupied the whole cell diameter (Suppl Fig. 2D). Ligation of the internodal cells is a serious damage, which is always followed by the deposition of a wound wall (Foissner and Wasteneys 2012). As described above, wound walls had also been deposited beneath the plasmodesmata-containing cell walls following destruction of the neighbor cell. The surface of these wound walls was smooth, and charasomes were abundant along their surface (Figs. 2i–k and 5). We therefore argued that the morphology of the wound wall could be decisive for the reported alkalinization and for the presence or absence of charasomes. In the following, we describe pH patterns and charasomes at wound walls with uneven surfaces that protrude deep into the streaming endoplasm. The results were similar in the internodal cells of the main axis and in the internodal cells of the branchlets and whether internodal cells were wounded at acid or at alkaline regions.

**Fig. 4:**
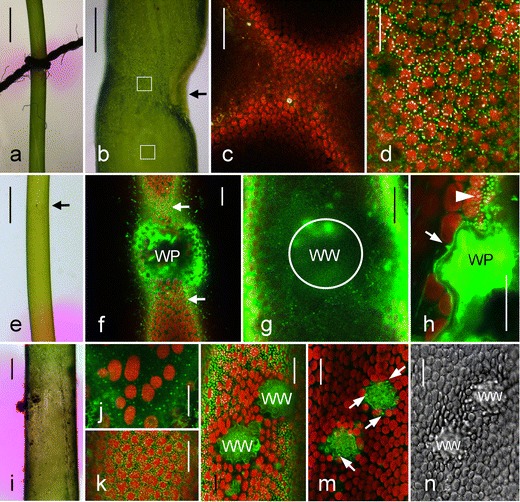
Charasomes are absent from uneven wound walls. **a**–**d** Ligation-induced wound walls. The medium near the ligation site of an internodal cell has an alkaline pH (*pink*) as visualized by phenol red. Cell deformation and a thick wound wall (*arrow*) becomes visible after removal of the thread (**b**). Insets correspond to the images (**c**) and (**d**). Charasomes (*green* FM1-43-fluorescent) are absent from the ligation site ((**c**), corresponding to the upper inset in (**b**)) and present between *red fluorescent* chloroplasts in the adjacent acidic control region ((**d)**, corresponding to the lower inset in (**b**)). **e**–**k** Wound walls (*WW*) induced by puncturing. A single puncture wound (*arrow*) located within an acidic region is seen in (**e**). The optical section through the periphery of the cell shows abundant FM1-43 fluorescent charasomes (*arrows*) outside the wound plug (*WP*) which seals the cell wall hole (**f**). No charasomes are visible in an optical section along the wound wall (*encircled* in (**g**)). *Green fluorescent particles* outside the wound wall are mobile FM1-43-stained organelles (putative endosomes) in the endoplasm. The *arrow* in the optical longitudinal section through a wound plug in (**h**) points to a single charasome at the otherwise smooth plasma membrane along the non-fluorescent wound wall. Charasomes are seen between the *red fluorescent* chloroplasts in the unwounded area (*arrow head*). Multiple puncture wounds cause an alkalinization of the phenol red-containing medium (**i**). Few charasomes (*green fluorescent* after staining with FM1-43) are seen in the wound area (**j**) in comparison with the unwounded region (**k**). **l**–**n** Wound walls caused by epiphytes. The optical section through the cortical cytoplasm shows numerous FM1-43-stained charasomes (*green*) between *red fluorescent* chloroplasts surrounding the wound walls (**l**). Only few charasomes (*arrows* in (**m**)) are present at the inner surface of the wound walls; corresponding DIC image (*N*). *Bars* = 1 mm (**a**, **e**), 300 μm (**i**), 150 μm (**b**) 50 μm (**c**), 20 μm (**d**, **f**, **g**, **j**-**n**), 10 μm (**h**)

We first repeated the ligation experiments and studied pH banding and charasome distribution in response to artificial constriction of the cell. The internodal cells were exposed to the air until turgor was lost and ligated at an acid band with the aid of cotton thread (Fig. 4a, Shimmen and Yamamoto 2002). Cells were allowed to recover in artificial fresh water, and the pH banding pattern in medium supplemented with phenol red was recorded over a period of several weeks. The pH at ligated regions was consistently alkaline if ligation was strong enough to induce the deposition of a thick wound wall which protruded deep into the endoplasm and eventually caused deformations in cell shape (Fig. 4a, b). In our hands, chloroplasts mostly remained in place, but charasomes were absent from the ligation-induced wound walls as revealed by CLSM of FM1-43-stained cells 2 weeks after injury (Fig. 4c). The charasome size and abundance in the adjacent unwounded acid regions were as in control cells (Fig. 4d). Cotton threads which were only slightly constricted and caused no visible effect on cytoarchitecture did not lead to alkalinization of the medium, and this experiment also excluded that local shading by the cotton thread caused charasome degradation.

**Fig. 5:**
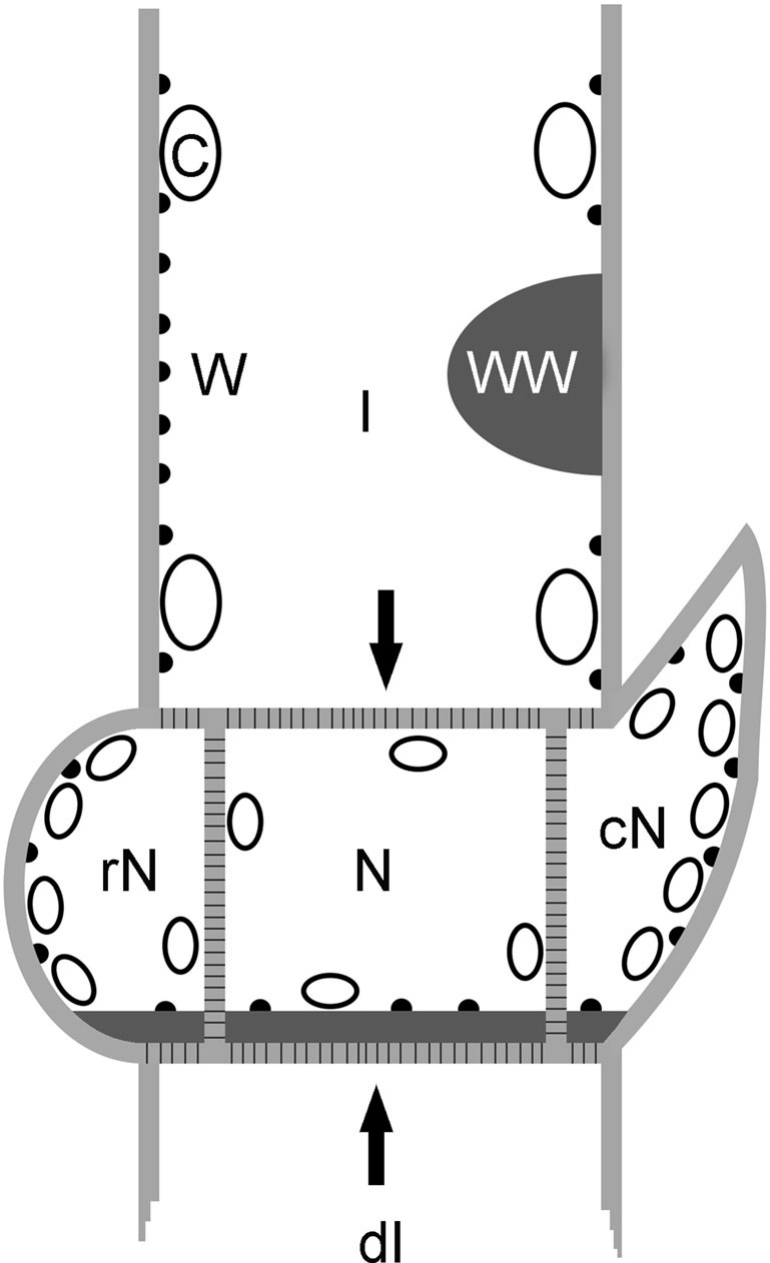
Schematic summary. In intact internodal cells (*I*) charasomes (*black dots*) are present between chloroplasts (*C*) at the longitudinal cell walls but absent from the chloroplast-free cross walls (*arrows*) which are perforated by plasmodesmata-containing channels (*short black lines* within the *light gray cell wall*). Charasomes may form at chloroplast-free windows (*W*) but they are absent from uneven wound walls (*WW*) which protrude into the endoplasm (not drawn). In nodes charasomes are present along the outer cell walls of peripheral, roundish nodal cells (*rN*), and of peripheral, cone-shaped nodal cells (*cN*; bract cells or stipulodes). No charasomes are found beneath the plasmodesmata-containing cell walls. Following damage of internodal cell (*dI*), a plasmodesmata-free wound wall (*dark gray line*) is deposited by the adjacent nodal cells (*N*) and charasomes may form along its smooth surface. Only approximately drawn to scale and with reduced number of nodal cells

We then investigated the wound walls formed after perforating the cell wall with the aid of tungsten needles (Foissner 1988). Puncture wounds were sealed by vacuolar material and damaged cytoplasm, including chloroplasts, which were expelled due to the turgor of cells. Onto this wound plug, which also contains membranous material stained by FM1-43, a thick cellulosic wound wall was deposited (Fig. 4e–h; Klima and Foissner 2011). Wound plug and wound wall protruded deep into the endoplasm (Figs. 4h and 5). The plasma membrane beneath the wound wall of cells investigated 2 weeks after puncturing was largely smooth even if charasomes were abundant in the surrounding cytoplasm (Fig. 4h). Puncturing at acidic domains led to a transient increase in local pH for several minutes as described for microperforated cells (Bulychev and Komarova 2014b) and as also observed during window formation (see above), but long-term monitoring never revealed a permanent alkalinization (Fig. 4e; seven of ten puncture wounds in branchlet internodes remained acidic, and the remaining three puncture wounds became transiently alkaline). Puncture-induced wound walls measured up to 50 μm in diameter and were therefore negligible small as compared with the size of the internodal cells (Fig. 4e). We therefore assume that the reduced number or absence of charasomes at small, delicate wounds caused slight alkalinization (or less acidification) of cell surface, but the pH differences were not large enough to produce a color change in phenol red. Consistent with this interpretation, we found that the application of multiple puncture wounds close to each other were more likely to induce permanent alkalinization (Fig. 4i; seven of ten puncture wounds became permanently alkaline).

The internodal cells may become several months and even several years old (own unpublished observations). Older cells often contained distinct local thickenings of the cell wall which were probably induced by epiphytes, e. g., by fungal hyphae trying to penetrate the cell wall. The wall ingrowths displaced the cortical chloroplasts and protruded deep into the streaming endoplasm (Fig. 4l–n) just as the wound wall beneath the puncture wounds. Only few, if any, charasomes were present at the plasma membrane along these wall ingrowths even if they were abundant between the chloroplasts of the adjacent regions.

## Discussion

### Photosynthesis-dependent formation of charasomes is not restricted to the internodal cells

The convoluted plasma membrane areas of characean algae have hitherto been only described from the internodal cells of the main axis and of the branches and from the internodal cells of the branchlets (see introduction). During the course of this study, we also detected charasomes in the internodal cells of protonemata and in the internodal cells of rhizoids when they developed chloroplasts following light exposure. Even more interestingly, charasomes were found in chloroplast-containing nodal cells, irrespective whether chloroplasts were anchored in the cortex or participated in cytoplasmic streaming (Fig. 5). Charasomes were never detected in pale cells lacking well-developed chloroplasts consistent with the observation that photosynthesis is required for charasome development (Bisson et al. 1991; Chau et al. 1994; Schmoelzer et al. 2011). Apart from this prerequisite, every cell of the vegetative part of the thallus of *Chara australis* seems to have the capacity for charasome formation, although to different extent.

### Charasome formation is not dependent on close contact with chloroplasts or mitochondria

In the internodal cells, charasomes usually develop in close proximity to the stationary chloroplasts, and they are absent from the chloroplast-free neutral line and the chloroplast-free cross walls as confirmed in this study. Therefore, it had been suggested that charasome formation is dependent on close contact with chloroplasts, and that the removal of chloroplasts induced the formation of (charasome-free) alkaline band in mechanically wounded cells (Shimmen and Yamamoto 2002). We tested this hypothesis by analyzing the chloroplast-free regions, “windows”, experimentally induced by irradiation with intense blue light. The results of this study showed that the pH banding pattern was completely independent of the position of chloroplasts or windows, respectively. The fact that the pH at windows could be either acidic or alkaline indicates that anchored chloroplasts are neither required for extrusion of OH^−^ (or proton influx, Shimmen and Yamamoto 2002) nor for the acidification of the surrounding medium. Corresponding to the pH banding pattern, charasome distribution was found to be independent of the position of cortical chloroplasts. Even more interestingly, we found that charasome density could be higher in the chloroplast-free windows than that in the adjacent chloroplast-containing regions of the cell suggesting that the presence of chloroplasts anchored to or near the cell membrane restricted charasome growth due to space constraints at least under the conditions used in this study, i. e., after 2 weeks of growth of charasomes at rather low light intensity. We have to note, however, that after longer treatment or under higher light intensities, charasomes form not only between chloroplasts but also beneath them (Franceschi and Lucas 1980; Schmoelzer et al. 2011).

Our data indicate that charasome formation—although dependent on photosynthesis—does not require intimate contact of chloroplasts with the plasma membrane. Obviously, long-distance signaling from chloroplasts to the plasma membrane is sufficient to induce its invagination into complex domains. A possible candidate is H_2_O_2_ which has recently been shown to be transported from illuminated to non-illuminated regions of the internodal cells by cytoplasmic mass streaming (Eremin et al. 2013; Bulychev and Komarova 2014a). The shift in cytoplasmic redox poise and light-induced elevation of cytoplasmic pH appeared to be involved in the generation of the pH banding pattern by the opening of H^+^/OH^−^ permeable channels in the plasma membrane at the downstream region of the irradiated zone (Eremin et al. 2013). We speculate that the pH banding pattern, which precedes charasome formation (Chau et al. 1994), affects the properties of the plasma membrane and the cortical cytoplasm. It is feasible that endocytosis becomes locally inhibited at acidic zones so that surplus membrane area delivered by exocytosis of vesicles becomes convoluted into charasomes. This in turn would lead to a stronger acidification because of the highly concentrated H^+^-ATPases. Research is now in progress how local differences in external and internal pH affect charasome formation.

In spite of the slowly changing pH banding pattern, charasome size and abundance largely correlated with the positions of acid and alkaline zones investigated during the course of this study. Hence, charasomes became degraded and were newly formed in untreated cells or cell regions as long as their cell walls were not calcified. This is consistent with our observations that charasomes were often found in windows which were produced at the previously charasome-free alkaline regions and, on the other hand, charasomes were often found to be absent from windows produced at the previously charasome-rich acidic zones. More rapidly changing pH banding patterns (within few hours) have been described elsewhere, and it is unlikely that they correspond to the distribution of charasomes because of their slow growth (e.g., Bisson et al. 1991).

In the cortex of the unwounded control internodal cells, charasome size and density were shown to correlate with the size and density of mitochondria (Franceschi and Lucas 1980; Schmoelzer et al. 2011). At the acid regions, charasomes and also mitochondria were abundant, squeezed between the chloroplasts and in close contact to each other (Schmoelzer et al. 2011). In the cortical cytoplasm of windows, mitochondria were less frequently observed than in the adjacent chloroplast-containing regions where they were shielded from endoplasmic flow. Mitochondria occasionally adhered to the cortex but mostly detached after several seconds or minutes and were carried away with the streaming endoplasm. In the cortex of untreated internodal cells, mitochondria move along actin filaments and microtubules (Foissner 2004). Preliminary results indicated, however, that their anchorage in windows was actin and microtubule independent. Irrespective of how mitochondria adhered to windows, the forces between cortex and mitochondria were obviously weaker than those exerted by the shearing forces of cytoplasmic mass streaming. It also became clear from these observations that contact with mitochondria was not required for charasome growth.

### Do functional plasmodesmata inhibit charasome formation?

Our study confirmed that charasomes were absent from the chloroplast-free transverse end walls of the internodal cells (Franceschi and Lucas 1980). These cell walls were pervaded by plasmodesmata which connected the cytoplasm of the internode with that of the adjacent nodal cells (Brecknock et al. 2011; Cook et al. 1997). Charasomes were also absent from the plasmodesmata-containing cell walls between different nodal cells. When plasmodesmata became severed due to the destruction of the neighboring internodal cell, a continuous, smooth, and channel-free cell wall was deposited onto the old plasmodesmata-containing cell wall. The plasma membrane along this newly formed “wound wall”, which had a smooth surface, was able to invaginate into charasomes (Fig. 5). These findings suggest that the presence of functional plasmodesmata, a highly complex membrane macrodomain (Boutte and Moreau 2014), excludes the presence of another complex membrane domain, the charasome. Although we cannot exclude that the absence of charasomes is due to sterical restrictions, it is feasible that the presence of plasmodesmata-associated proteins prevents charasome formation. That *Chara* plasmodesmata, like those of higher plants, contain a specific set of proteins, has been documented (Faulkner et al. 2005). However, it is also possible that charasomes can only be formed when the cell wall is directly exposed to the environment. Studies are now in progress to investigate which of these two possibilities, or both, are responsible for charasome development.

In nodal cells, the presence of functional plasmodesmata does not prevent the anchoring of chloroplasts. The absence of chloroplasts at the end walls of the internodal cell is therefore due to other constraints. A likely candidate is the actin cytoskeleton which, in co-operation with myosin, drives cytoplasmic mass streaming. Actin filaments are usually polymerized from the plasma membrane. The subcortical actin filament bundles in the internodal cells are aligned along the chloroplast files, far apart from the plasma membrane. If chloroplasts are not tightly packed, the subcortical actin bundles may extend into the cortex. Otherwise, close contact of actin filament bundles with the plasma membrane is possible only at the end walls, and it is feasible that these regions are free of chloroplasts (and charasomes) in order to accommodate protein complexes involved in actin polymerization and anchorage (e. g., Martiniere et al. 2011; Thomas 2012). The presence of chloroplasts at the plasmodesmata-containing cell walls of nodal cells with a streaming endoplasm can be explained by the fact that chloroplasts of the nodal cells are smaller and wider spaced so that there is ample space for anchoring or nucleation of actin filaments between chloroplasts.

### pH banding pattern and charasome formation at wound walls

We confirmed that alkaline bands form at ligated regions of the internodal cells (Shimmen and Yamamoto 2002) and showed that the plasma membrane beneath the ligated zone was free of charasomes as expected. However, this study also revealed that the local removal of chloroplasts did not lead to local, permanent alkalinization of the cell surface and, furthermore, ligation was not always associated with the detachment of chloroplasts. This excludes the possibility that the absence of chloroplasts is responsible for the local and permanent rise in pH. During the course of this study, we observed that alkaline (charasome-free) zones were created after the deposition of extended wound walls with an uneven surface, irrespective whether they were induced by ligation or by multiple puncturing. We also found that charasomes were absent beneath wounds induced by single puncturing or by epiphytes. Both wound types were characterized by uneven wound walls which protruded deep into the endoplasm and both were (nearly) devoid of charasomes (Fig. 5). We therefore hypothesize that the absence of charasomes along bulged wound walls is due to hydrodynamic turbulences in the streaming endoplasm (Uijttewaal 2014). Such turbulences do not occur along smooth surfaces, and this would explain why numerous charasomes were observed along the flat, smooth wound walls deposited beneath plugged plasmodesmata.

## Electronic supplementary material

Below is the link to the electronic supplementary material.

Suppl. Video 1 Nodal cell of *Chara australis* with motile chloroplasts (autofluorescent red) and minute FM1-43 fluorescent charasomes (green; arrows). Time interval is 2580 ms; 5 frames per second. (AVI 842 kb)

Suppl. Fig. 1 Charasomes visualized by FM1-43 in branchlet internodal cells of *Chara australis* treated with pH buffers and with vanadate, an inhibitor of H^+^-ATPases for one hour. **a** Cell treated with 2 mM MES (pH 5.5). **b** Cell treated with 2 mM TAPS (pH 9). **c** Cell treated with 5 mM vanadate. All cells were taken from the same whorl to ensure homogeneity of the material. All treatments inhibited pH banding; images were taken at the previously acid regions. (JPEG 0.98 mb)

Suppl. Fig. 2 pH banding patterns and windows in internodal cells of the main axis of *Chara australis*. **a, b** Schematic drawings of the pH banding patterns in a cell without windows (**a**) and in a cell with two small and two large windows (white circles in (**b**)). The pH was documented in one week intervals using phenol red; pink regions correspond to alkaline pH. **c, d** Light micrographs of internodal cells with large windows and incubated in phenol red. In (**c**) two small and two large windows were created on opposite sides of the cell. Note different pH in spite of similar dimensions. The belt-shaped acid window in (**d**) runs across the circumference of the cell. Bars = 2 mm (**a, b**) and 1 mm (**c, d**) (JPEG 867 kb)
