# Supplementary figures and images for: Photosynthesis-dependent formation of convoluted plasma membrane domains in Chara internodal cells is independent of chloroplast position
Source: Protoplasma. 2014 Dec 19;252(4):1085–96. doi: 10.1007/s00709-014-0742-9 (PMC4493373; doi:10.1007/s00709-014-0742-9)

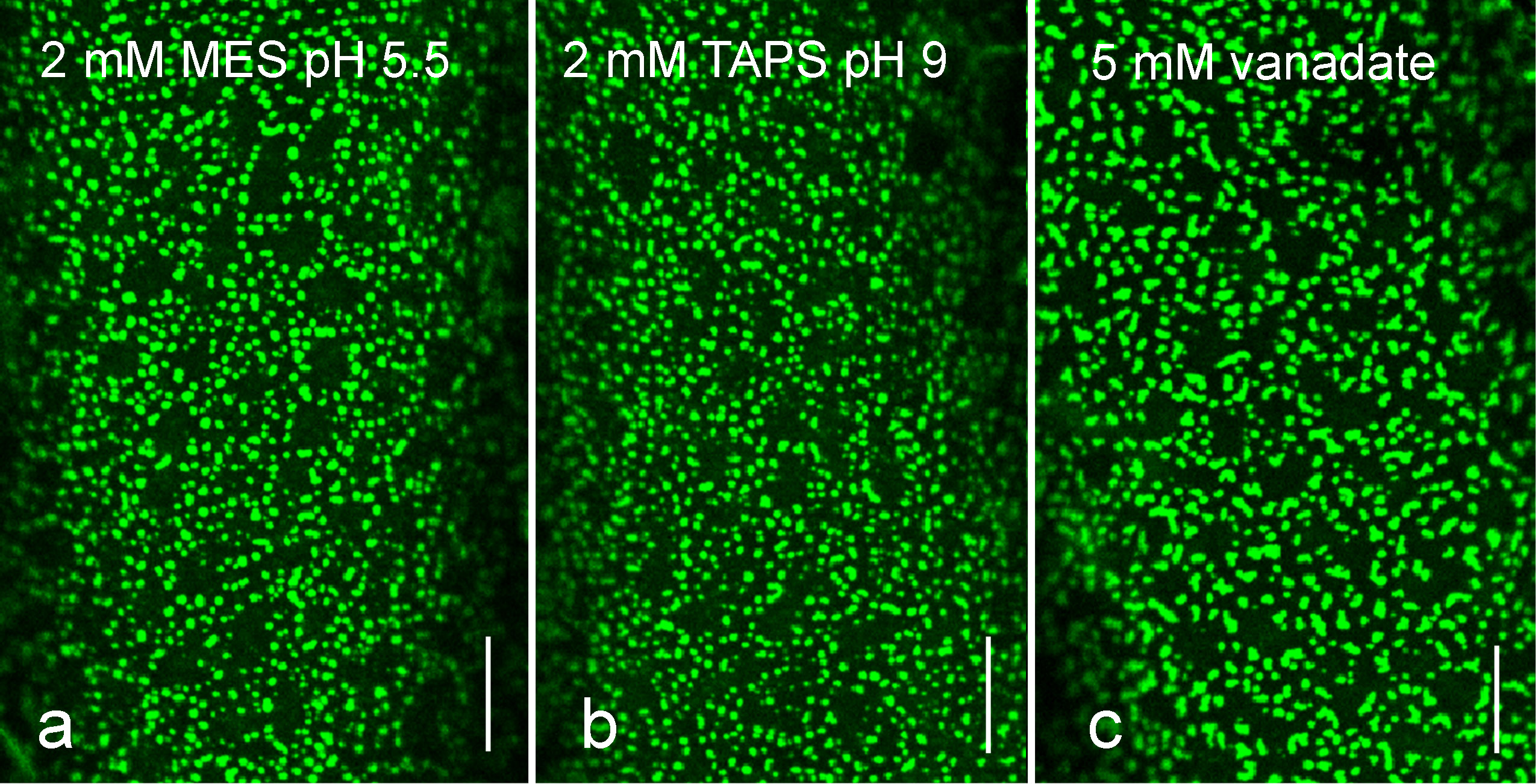

Supplement: Supplementary file 2 — Charasomes visualized by FM1-43 in branchlet internodal cells of Chara australis treated with pH buffers and with vanadate, an inhibitor of H+-ATPases for one hour. a Cell treated with 2 mM MES (pH 5.5). b Cell treated with 2 mM TAPS (pH 9). c Cell treated with 5 mM vanadate. All cells were taken from the same whorl to ensure homogeneity of the material. All treatments inhibited pH banding; images were taken at the previously acid regions. (JPEG 0.98 mb) [file 709_2014_742_Fig6_ESM.jpg]

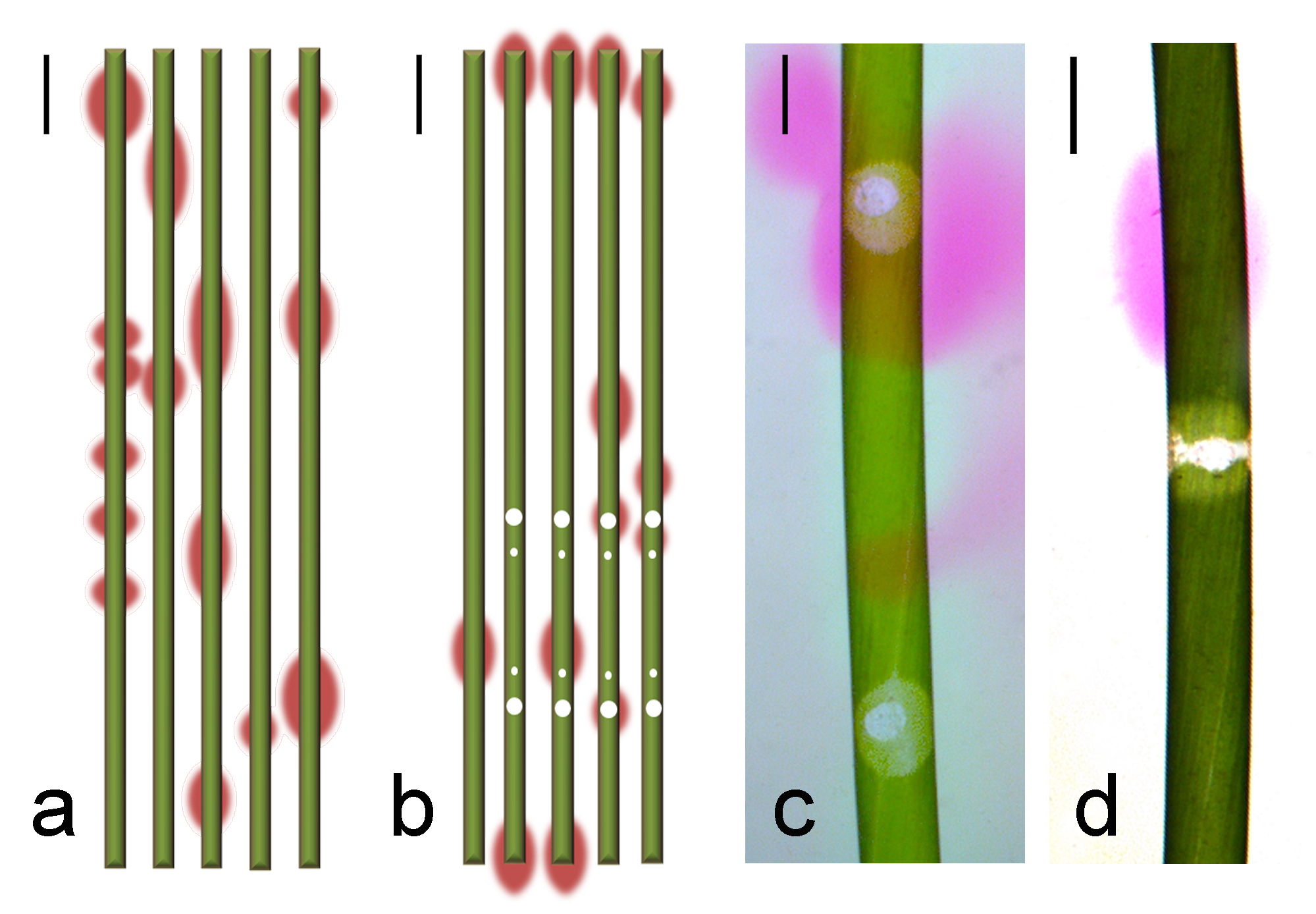

Supplement: Supplementary file 3 — pH banding patterns and windows in internodal cells of the main axis of Chara australis. a, b Schematic drawings of the pH banding patterns in a cell without windows (a) and in a cell with two small and two large windows (white circles in (b)). The pH was documented in one week intervals using phenol red; pink regions correspond to alkaline pH. c, d Light micrographs of internodal cells with large windows and incubated in phenol red. In (c) two small and two large windows were created on opposite sides of the cell. Note different pH in spite of similar dimensions. The belt-shaped acid window in (d) runs across the circumference of the cell. Bars = 2 mm (a, b) and 1 mm (c, d) (JPEG 867 kb) [file 709_2014_742_Fig7_ESM.jpg]
